# Effect of Calcination Temperature on the Activity of Unsupported IrO_2_ Electrocatalysts for the Oxygen Evolution Reaction in Polymer Electrolyte Membrane Water Electrolyzers

**DOI:** 10.3390/molecules28155827

**Published:** 2023-08-02

**Authors:** Angeliki Banti, Kalliopi Maria Papazisi, Stella Balomenou, Dimitrios Tsiplakides

**Affiliations:** 1 Physical Chemistry Laboratory, Chemistry Department, Aristotle University of Thessaloniki, 541 24 Thessaloniki, Greece; 2 Chemical Process and Energy Resources Institute, Centre for Research and Technology Hellas, 570 01 Thessaloniki, Greece; papazisi@certh.gr (K.M.P.); stellab@certh.gr (S.B.)

**Keywords:** iridium oxide, oxygen evolution, PEM water electrolyzer

## Abstract

Polymer electrolyte membrane (PEM) water electrolyzers suffer mainly from slow kinetics regarding the oxygen evolution reaction (OER). Noble metal oxides, like IrO_2_ and RuO_2_, are generally more active for OER than metal electrodes, exhibiting low anodic overpotentials and high catalytic activity. However, issues like electrocatalyst stability under continuous operation and cost minimization through a reduction in the catalyst loading are of great importance to the research community. In this study, unsupported IrO_2_ of various particle sizes (different calcination temperatures) were evaluated for the OER and as anode electrodes for PEM water electrolyzers. The electrocatalysts were synthesized by the modified Adams method, and the effect of calcination temperature on the properties of IrO_2_ electrocatalysts is investigated. Physicochemical characterization was conducted using X-ray diffraction (XRD), Brunauer–Emmett–Teller (BET) surface area measurement, high-resolution transmission electron microscopy (TEM), and X-ray photoelectron spectroscopy (XPS) analyses. For the electrochemical performance of synthesized electrocatalysts in the OER, cyclic voltammetry (CV) and linear sweep voltammetry (LSV) were conducted in a typical three-cell electrode configuration, using glassy carbon as the working electrode, which the synthesized electrocatalysts were cast on in a 0.5 M H_2_SO_4_ solution. The materials, as anode PEM water electrolysis electrodes, were further evaluated in a typical electrolytic cell using a Nafion^®^115 membrane as the electrolyte and Pt/C as the cathode electrocatalyst. The IrO_2_ electrocatalyst calcined at 400 °C shows high crystallinity with a 1.24 nm particle size, a high specific surface area (185 m^2^ g^−1^), and a high activity of 177 mA cm^−2^ at 1.8 V for PEM water electrolysis.

## 1. Introduction

Hydrogen, owing to its high mass–energy density (the highest of any fuel) and its clean combustion in air, may be considered the ultimate clean energy carrier. Hydrogen can be produced through water electrolysis using surplus electricity from variable renewable energy sources and thus become a solution for storing electricity, which can be further used as a carbon-free fuel. Among the different electrolysis technologies, proton-exchange membrane water electrolyzers (PEMWEs) are one of the most attractive and efficient technologies for water electrolysis due to (i) wide-operational range of current densities; (ii) high energy efficiency (40–50%); (iii) excellent dynamic response to variable input and on/off cycling; (iv) the ability to operate at high operating pressure values; (v) high-purity hydrogen production; and (vi) safety, non-pollution, and long lifetimes [1,2].

In a PEMWE, liquid water is generally introduced in the electrolysis cell from the anode side, where it passes through the anode porous current collector and reaches the anode electrocatalyst by enriching the polymer membrane as well. Water is electrolyzed (oxidized) towards the production of H^+^ and molecular O_2_ in the anode side (oxygen evolution reaction, OER). Proton ions pass through the polymer electrolyte membrane and reach the cathode electrocatalyst, where their reduction takes place toward the production of molecular H_2_ (hydrogen evolution reaction, HER). In conventional PEM water electrolysis cells, platinum black (unsupported Pt particles) is used as an electrocatalyst at the cathode for HER, as it takes place with low overpotential values. In contrast, the OER involves a much more complex mechanism, resulting in slow kinetics and high electrode overpotentials [3]. Studies have been extensively conducted in order to mitigate the drawbacks of OER irreversibility and slowness, as well as to find a reversible electrocatalyst, resistant and chemically stable. Recently, a universal activity descriptor (orbital charge-transfer energy) and a unifying mechanism concept for both HER and OER have been proposed to guide the rational design of electrocatalysts and help avoid time-consuming and high-cost trials and errors in fundamental research and commercialization [4].

Various precious metals have been tested, such as Pt, Rh, Ru, Ir, and Ni [5]. However, oxides of each catalyst for the OER seem to have a dominant factor in affecting their electrocatalytic activities. Among the transition metal oxides, RuO_2_ and IrO_2_ exhibit high metallic conductivity and efficiency. A major drawback of RuO_2_ is that it is sensitive to corrosion and lacks stability. Besides Ru, Ir is the metal that exhibits the best catalytic properties for the OER compared to other noble metals, giving rise to a great number of studies on PEM water electrolysis concentrated on Ir-based catalysts and particularly pure IrO_2_ which is usually employed as an OER catalyst with typical loadings of 2–4 mg·cm^−2^ [6]. Based on the needed amount of PGMs, the cost of selected electrocatalysts represents a great portion of the MEA cost and, subsequently, the electrolyzer cost, and it remains a significant challenge to overcome for the technology to enter a commercial scale. Based on this, strong efforts are needed in order to make the process cost-effective and industrially attractive.

The synthesis of IrO_2_ nanoparticles with particle sizes ranging down to ~2 nm has been shown to effectively increase the number of surface atoms with respect to bulk and thus increase the OER activity per gram of catalyst [7]. A major issue that arises from this approach is that it becomes increasingly difficult to establish structure-activity relationships for the OER due to the complex nature of the surface species [8]. Towards this direction, several preparation methods have been reported for the synthesis of nano-sized IrO_x_ electrocatalysts, including the Adams method [9], the sol–gel method [10], the sulfite complex method [11], the magnetron sputtering method [12], the sonochemical method [13], and the template method [14]. Among them, the Adams method, first introduced by Adams and Shriner [15], presents several advantages being a simple synthesized process producing small, nano-scale particle size and high surface area catalysts [2]. Following the Adams method, nano-sized IrO_2_ has been synthesized with a crystallite size varying between 4 and 9 nm and a BET surface area in the range of 68–113 m^2^ g^−1^, depending on calcination temperature [7,16,17].

The effect of calcination temperature on the OER performance of IrO_2_ was investigated by Reier et al. [18] for IrO_2_ thin films prepared by the thermal decomposition of iridium Ir chloride precursors and by Silva et al. [3] for IrO_2_ synthesized according to the hydrothermal method. Both studies revealed that there is a strong relationship between structural properties and electrocatalytic performance/stability, which can be attributed to the presence of a mixture of amorphous and crystalline IrO_2_. Specifically, the high crystallinity of high-temperature Ir oxide species is detrimental, whereas low-temperature amorphous Ir oxyhydroxides are highly active and efficient catalysts for the OER. On the other hand, higher stabilities were achieved for the materials with increased crystallinity. In order to reveal the effect of calcination temperature on IrO_x_ activity and stability for OER, online inductively coupled plasma mass spectrometry (ICP-MS) experiments were conducted by Cherevko et al. [19]. They concluded that the stability and OER activity of the IrO_x_ catalysts strongly depend on the chemical and structural nature of the Ir oxide species and their synthesis conditions. Specifically, the calcination temperature resulting in different crystallinity of the IrO_2_ film affects the Ir dissolution rate, with the stability essentially increasing with increasing calcination temperature and crystallinity. Therefore, regardless of the IrO_x_ synthesis method, most studies agree that regarding the activity of the OER, amorphous iridium oxide is regarded to be more active than the crystalline version, while the latter is considered to be more stable.

In this study, nano-sized unsupported IrO_2_ electrocatalysts with high specific surface area and high OER activity have been synthesized by the modified Adams method [15]. IrO_2_ electrocatalysts were calcined at 400, 500, and 600 °C and systematically studied the effect of calcination temperature on the morphology, structure, and performance. The catalysts were characterized using several techniques, such as specific surface area (BET) analysis, energy dispersive X-ray diffraction (XRD), X-ray photoelectron spectroscopy (XPS), and transmission electron microscopy (TEM). The prepared materials were electrochemically characterized, and their OER activities, as well as stabilities, were evaluated in half-cell configurations in an acidic electrolyte. The synthesized materials were used as anode electrocatalysts to fabricate membrane electrode assemblies (MEAs), which were studied in a single-cell electrolysis configuration regarding their activity and short-term stability.

## 2. Results

### 2.1. Structural Characterization of the Catalysts

**Figure 1:**
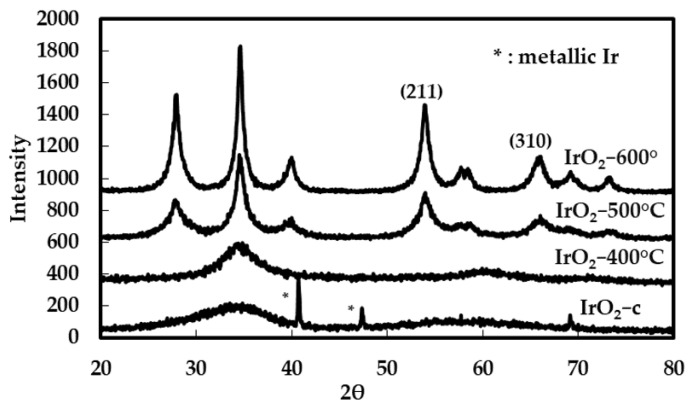
XRD patterns of IrO_2_-400 °C, IrO_2_-500 °C, IrO_2_-600 °C, and IrO_2_-c powder electrocatalysts.

XRD analysis was used to investigate the evolution in the crystalline structure of the unsupported catalysts with increasing calcination temperature. The XRD analysis was performed on the calcined catalysts (referred to as “IrO_2_-x °C”, where x is the sintering temperature) and on the commercial catalyst (“IrO_2_-c”). The corresponding diffraction patterns are shown in Figure 1. In the commercial and the IrO_2_-400 °C catalyst, two broad peaks (2θ = 35 and 59°) are clearly visible in both XRD patterns. The XRD results for these catalysts are characteristic of materials that are amorphous and/or have small-sized crystallites, while in the case of IrO_2_-c, some additional sharp peaks appear (2θ = 40.64, 47.36, and 69°), which are assigned to the metallic Ir. The characteristic peaks of the rutile tetragonal IrO_2_ started to develop in the diffraction patterns of the catalysts calcined at 500 °C and the peaks were fully developed in the diffraction patterns of catalyst calcined at 600 °C. In addition, by increasing the calcination temperature to 500 °C, the amorphicity of the electrocatalyst was observed to decrease. After raising the temperature to 600 °C, these reflection peaks became thinner and sharper, indicating a higher degree of crystallinity for this material. The XRD pattern also confirmed the absence of other phases, such as metallic iridium, which is present in the commercial IrO_2_ catalyst.

The average crystal size and the lattice parameter for each IrO_2_ electrocatalyst were calculated through the Scherrer equation. Specifically, the (101) reflection peak at 2θ = 36° was used to define the full width at half maximum (FWHM) intensity and to calculate the crystallite size of IrO_2_. Two different behaviors were observed. The XRD spectra of the IrO_2_-400 °C and IrO_2_-c materials present only small and broad diffraction peaks, while well-defined diffraction peaks are missing, which is a typical characteristic of amorphous catalysts. Consequently, the absence of defined diffraction peaks makes it impossible to use the Debye–Scherrer analysis, which meant that the mean crystallite sizes of the materials calcined at 400 °C, as well as the commercial catalyst, could not be determined. On the contrary, the materials calcined at higher temperatures, IrO_2_-500 °C and IrO_2_-600 °C, exhibited pronounced and resolved diffraction peaks that evolved with the increase in calcination temperature. For these catalysts, the mean crystallite size was determined to be 5.18 nm for the IrO_2_-500 °C, while that for the material obtained at 600 °C is 7.77 nm. Furthermore, the lattice parameters for all IrO_2_ catalysts were determined. The calculated parameters are summarized in Table 1, and they are in accordance with the JCPDS database for tetragonal IrO_2_-rutile crystallographic pattern (JCPDS No. 15-871). A slight increase in unit cell volume is observed for IrO_2_-600 °C compared to IrO_2_-500 °C (62.4 Å^3^ vs. 62.0 Å^3^), which implies the introduction of tensile strain and possible generation of oxygen vacancies. Theoretical computations reveal that this leads to the reduction in the thermodynamic energy barrier for hydroxyl adsorption and a concomitant improvement in alkaline OER on perovskite oxides [20]. Although the introduction of oxygen vacancies cannot be excluded for the rutile IrO_2_-500 °C and IrO_2_-600 °C, this is not expected to significantly affect their OER activity since hydroxyl adsorption is not a rate-determining step (rds) for any of the proposed reaction mechanisms regarding acidic OER [21]; however, they may affect performance stability.

**Figure 2:**
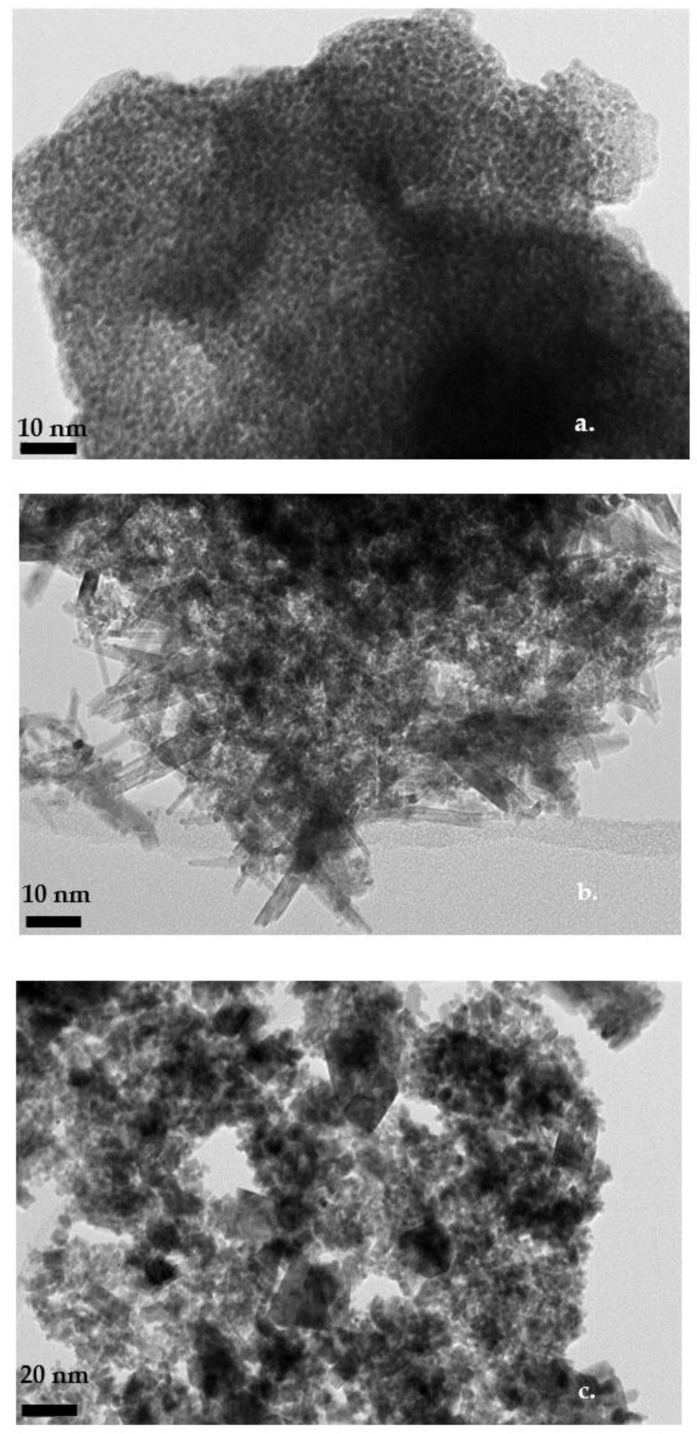
HRTEM images of IrO_2_ powders prepared under different calcination temperatures: (**a**) 400 °C, (**b**) 500 °C, and (**c**) 600 °C.

The transmission electron micrographs (TEM) for the IrO_2_ catalysts are presented in Figure 2a–c, following the synthesis of these catalysts and before the OER activity evaluation. As expected for unsupported catalysts, the TEM images revealed the presence of aggregates of different sizes composed of small nanoparticles, which is known to reduce catalyst utilization. Particularly, the TEM micrograph of the commercial and IrO_2_-400 °C catalysts reveal spherical particles with sizes in the 1–2 nm range; however, the mean particle size could not be safely determined due to the agglomeration of these nanoparticles. For the catalysts obtained at higher temperatures (Figure 2b,c), the spherical morphology is not retained; on the contrary, the particles appear in rectangular shape or as nanorods. Furthermore, the average particle size increases proportionately when the temperature rises to higher calcination temperatures because of thermal sintering. Specifically, the majority of particles for the IrO_2_ catalysts calcined at 500 °C and 600 °C are within the 5–7 nm range, in agreement with the XRD results (Table 1). The results confirm the effectiveness of the selected catalyst preparation method for the development of nano-sized materials with large surface areas.

**Figure 3:**
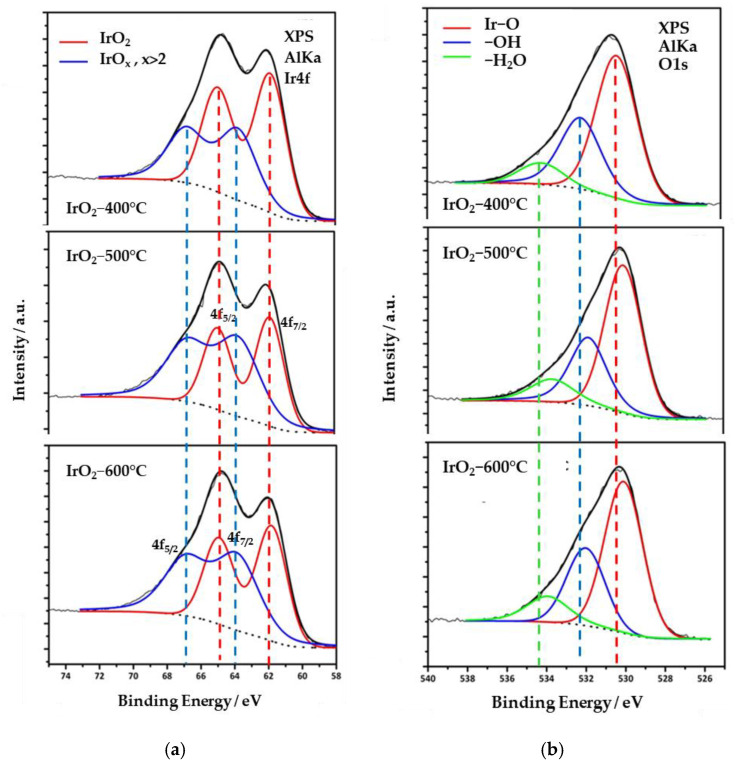
High-resolution XPS spectra corresponding to (**a**) Ir4f and (**b**) O1s orbital of the IrO_x_ electrocatalysts prepared under different calcination temperatures: 400 °C (**top**), 500 °C (**middle**), and 600 °C (**bottom**).

The chemical state of Ir in the catalysts was examined by XPS analysis. Figure 3a displays measurements of the Ir 4f orbital of all electrocatalysts. The deconvolution of each Ir 4f spectrum gives two sets of doublets, corresponding to 7/2 and 5/2 spin-orbit components. One doublet (red curve) is located at 61.9 (Ir 4f_7/2_) and 65.1 eV (Ir 4f_5/2_), and it can be attributed to Ir^4+^ (IrO_2_). All spectra appear to have an almost equal contribution from another doublet (blue curve) at binding energies 63.8 and 66.9 eV, which is attributed to Ir at a higher oxidation state, i.e., IrO_x_ (x > 2). The presence of surface Ir in two oxidative states and the corresponding binding energy values are close to the reported ones for chemically produced IrO_2_ [22,23]. In addition, the XPS measurements did not show any peak corresponding to metallic iridium (Ir^0^), in agreement with the XRD data. The surface composition of the catalysts, expressed in % percentages of Ir^4+^, are 41, 55, and 52% for IrO_2_-400 °C, IrO_2_-500 °C, and IrO_2_-600 °C catalysts, respectively. It should be noted that the above values refer to the surface composition of the IrO_2_ catalysts, which in general, may significantly deviate from the bulk composition. Nevertheless, as electrochemical reactions take place on the surface, it is particularly important to consider the surface state of the catalysts in relation to their electrochemical behavior.

High-resolution oxygen 1s-orbital spectra were also acquired and are displayed in Figure 3b. Three species are identified at ~530.4 eV, 532.2 eV, and 534.2 eV bond energies, attributed to Ir–O and Ir–OH bonds as well as weakly adsorbed molecules of water on their surface, respectively. The latter species do not participate in the stoichiometry of the catalyst and are related to surface adsorbates due to exposure to the atmosphere. Furthermore, the spectra reveal that the O 1s orbital shifts to lower energy with increasing calcination temperature. This shift reflects an enhancement in the oxygen content of the oxide form compared to the oxyhydroxide [24,25].

The oxygen-to-iridium atomic ratios were calculated using the signal intensity from O 1s and Ir 4f spectra, normalized for the different element sensitivity factors. It was found that the O:Ir ratio is between 2.41 to 2.82 for all three samples, which is slightly higher than the theoretical value of 2 for IrO_2_, revealing again the co-existence of iridium oxides at higher oxidation states. Furthermore, the O:Ir ratio did not exhibit a specific trend with sintering temperature, indicating that thermal treatment did not result in an oxygen loss on the surface due to the conversion of Ir^4+^ into Ir^3+^.

The BET surface areas of the calcined catalysts were determined using nitrogen physisorption isotherms and the BET equation [22]. The measured BET surface area is characterized by high values and is reduced with calcination temperature. The BET surface area was 185 m^2^ g^−1^ for IrO_2_-400 °C, 127 m^2^ g^−1^ for IrO_2_-500 °C, and reduced to 50% of its initial value (66 m^2^ g^−1^) when the catalyst was calcined at 600 °C. Despite the calcination temperature, these values are higher than the reported values in the literature [7,23] (32 m^2^ g^−1^) for commercial IrO_2_. It is observed that the modified Adams method results in the formation of nanosized particles with high surface areas. In TEM images, it was shown that the iridium-based species grew with calcination temperature. Therefore, the reduction in the BET surface area was sharper when the calcination temperature was increased from 400 to 600 °C. This is in agreement with TEM images, where the increase in the size of iridium-based species was more evident when the calcination temperature was increased from 400 to 600 °C.

### 2.2. Surface Electrochemistry

**Figure 4:**
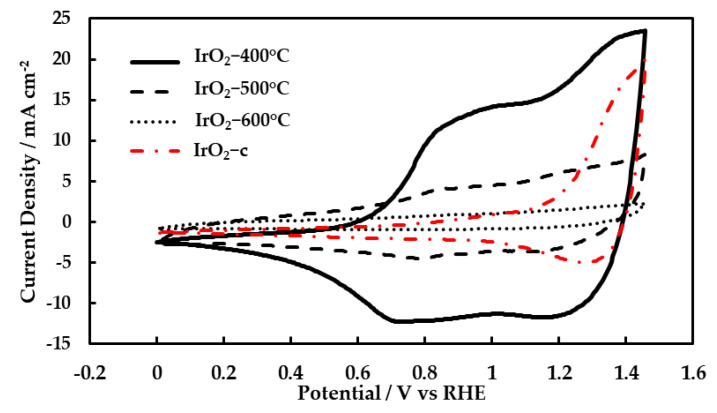
Cyclic voltammograms of IrO_2_-400 °C, IrO_2_-500 °C, IrO_2_-600 °C, and IrO_2_-c electrocatalysts at a scan rate of 20 mV s^−1^ in 0.5 M H_2_SO_4_ at 25 °C.

Cyclic voltammetry was performed on the calcined catalysts and the commercial IrO_2_. During the potential scan, the oxidation states of iridium may change, and thus well-defined current peaks can usually be identified on the studied electrodes. Figure 4 shows the last cycle of a series of cyclic voltammograms (CVs) for IrO_2_-400 °C, IrO_2_-500 °C, IrO_2_-600 °C, and IrO_2_-c catalysts recorded at 20mV s^−1^ scan rate in 0.5 M aqueous H_2_SO_4_ at room temperature. One well-defined oxidation reduction peak was observed at about 0.85 V vs. RHE (more pronounced for the IrO_2_-400 °C). This redox peak corresponds to the redox reaction of iridium, where the oxidation state changes between Ir^3+^ and Ir^4+^. Additionally, one reduction peak was observed at 1.30 V vs. RHE, which is attributed to the change in the Ir^4+^ to Ir^5+^ transition. The oxidation peaks for IrO_2_-500 °C, IrO_2_-600 °C, and IrO_2_-c catalysts corresponding to the above oxidation state transitions were not clearly observed. This was most likely due to an overlap with the OER. The OER starts around 1.43 V vs. RHE, where the oxidation peak was supposed to be seen. The recorded current for the CVs decreased with calcination temperature, and it was accompanied by less pronounced redox peaks and a smaller surface area under the voltammograms.

The surface area under the voltammograms divided by the scan rate corresponds to the voltammetric charge. The oxidation voltammetric charges were obtained from CVs over the whole anodic potential window. The charge transferred during the iridium redox reaction is proportional to the number of iridium active sites. It is frequently used as an estimation of electrochemically active surface area (ECSA). The ECSA reduced with the increase in calcination temperature (Table 2). This was due to the growth of iridium particles with calcination temperature. This result is comparable with the reported BET surface area results, where a similar trend was observed. According to the TEM images, 400–600 °C is the temperature range where a major increase in the size of iridium species takes place [26].

### 2.3. Activity for the Oxygen Evolution Reaction (OER)

**Figure 5:**
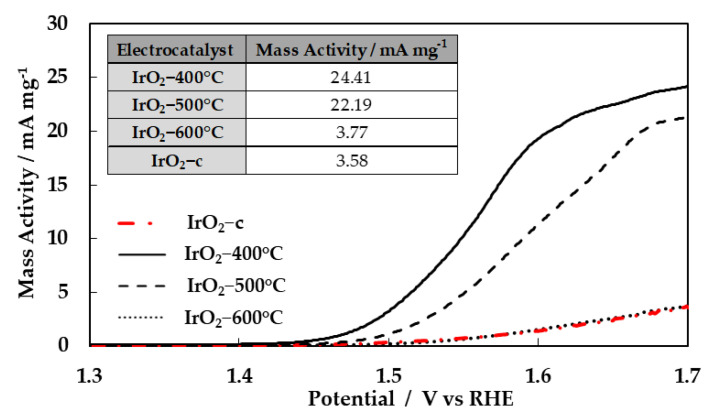
OER polarization curves at 1 mV s^−1^ of the IrO_x_ electrocatalysts in the potential range of 1.30 to 1.70 V vs. RHE in 0.5 M H_2_SO_4_. The currents have been normalized to express mass activity.

The catalysts’ activities toward the oxygen evolution reaction were evaluated through the polarization curves (LSV measurements), as shown in Figure 5. The currents in the curves are presented in the form of mass activity (mA mg^−1^ Ir). As depicted in this figure, the catalysts’ performance decreased as the calcination temperature increased. The performance of the commercial IrO_2_ is also measured and added to the figure for comparison. The mass activities at 1.70 V vs. RHE are summarized in the inset table (Figure 5). 

It is evident that the performance of the calcined catalyst (especially IrO_2_-400 °C) synthesized by the Adams method is superior to the commercial IrO_2_. The amorphous IrO_2_-400 °C catalyst is the most active, and its corresponding starting potential of oxygen evolution is the lowest. With the increase in calcination temperature, the starting potential of oxygen evolution increases since the IrO_2_-400 °C is more electrocatalytically active (more active sites) than IrO_2_-500 °C and IrO_2_-600 °C in OER, resulting in lower current densities at any potential. This decrease to the current (in the performance) with the increase in calcination temperature was due to the reduction in the available ECSA, as previously discussed. This is comparable with the result discussed in the surface electrochemistry section regarding cyclic voltammograms. Moreover, it could be concluded that the amorphous iridium-based species have higher activity toward OER compared to crystalline IrO_2_ [18,22].

**Figure 6:**
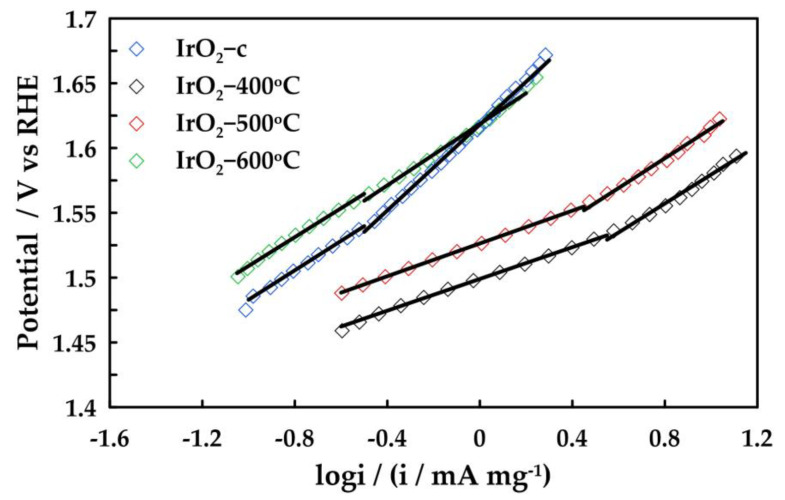
Tafel plots of ΙrO_2_-400 °C, ΙrO_2_-500 °C, ΙrO_2_-600 °C, and ΙrO_2_-c electrocatalysts.

To further investigate the OER activity of the catalysts, the OER Tafel slopes for the prepared IrO_2_ samples were evaluated in 0.5 M H_2_SO_4_. For this purpose, the corresponding Tafel plots are shown in Figure 6 for the voltage range of 1.40–1.65 V vs. RHE, and the calculated Tafel slope for each sample is listed in Table 3. The Tafel slope is an important kinetic parameter for revealing changes in the apparent OER mechanism.

Two distinguishable linear regions can be observed, with Tafel slopes close to 60 mV dec^−1^ in the low overpotential region and with Tafel slopes close to 115–118 mV dec^−1^ in the higher overpotential region. Tafel slopes of 40 and 120 mV dec^−1^ are common for OER at sputtered iridium oxide films and thermally prepared iridium oxides [18]. The resulting Tafel slopes at low overpotentials deviated slightly from 55 mV dec^−1^ obtained for anodically oxidized Ir nanoparticles and are in good accordance with 61 mV dec^−1^ obtained for thermally prepared IrO_2_ [27,28]. A Tafel slope of 60 mV dec^−1^ corresponds to an additional chemical step within the electrochemical oxide path in acidic media, which is the OH restructuring step (S–OH*_ads_ → S–OH_ads_, where S–OH*_ads_ and S–OH_ads_ stand for chemisorbed OH* species and reactive OH intermediates, respectively). This step follows the water discharge step (S + H_2_O → S–OH*_ads_ + H^+^ + e^−^, where S stands for the electroactive surface sites) and involves the activation of chemisorbed OH* groups to reactive OH species. However, at higher current densities, a Tafel slope of 120 mV dec^−1^ indicates that the rate-determining step moves to the oxidative adsorption of water [3]. Finally, Tafel slopes for ΙrO_2_-600 °C and ΙrO_2_-c are observed to be higher both of low and high overpotentials and are attributed to the formation of oxides on the surface which is in agreement with the results of XPS analysis.

**Figure 7:**
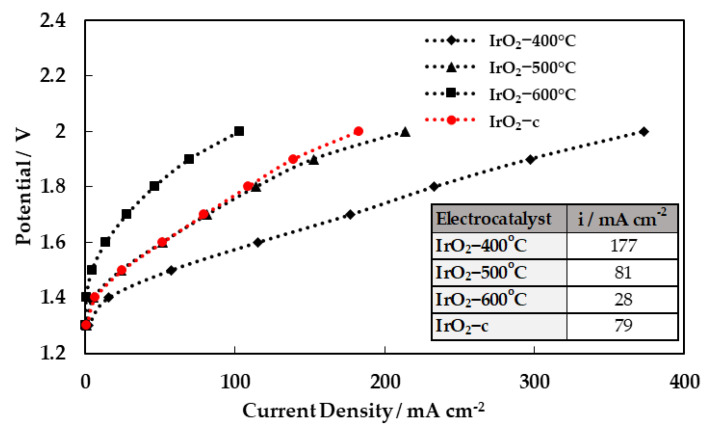
Performance curves (potential vs. current density) for the MEAs employing the four different IrO_2_ catalysts as an anode electrode. Inset table: current densities at 1.8 V.

A single-cell PEM water electrolysis cell was used to assess the performance of the synthesized IrO_2_ oxides under typical water electrolysis conditions. Each experiment was repeated three times and then characterized using polarization curves. All parameters (ionomer content, cathode, and membrane) were kept constant except the calcination temperature of the IrO_2_ catalyst. Electrolysis performances were measured at a constant temperature of 50 °C at atmospheric pressure (1 bar). Liquid water was fed at the anode with a mass flow rate of 300 g h^−1^, while He saturated with vapor (58 kPa H_2_O) was supplied at the cathode. The corresponding steady-state polarization curves for all catalyst formulations are shown in Figure 7. The cell with IrO_2_-400 °C as the anodic electrocatalyst exhibited the highest performance among all samples, achieving 177 mA cm^−2^ at 1.8 V. It should be noted that the reported values are not corrected for the ohmic losses. The superior performance of the MEA with the IrO_2_ calcined at 400 °C is in agreement with the findings of the intrinsic OER activity measurements performed in the RDE configuration.

**Figure 8:**
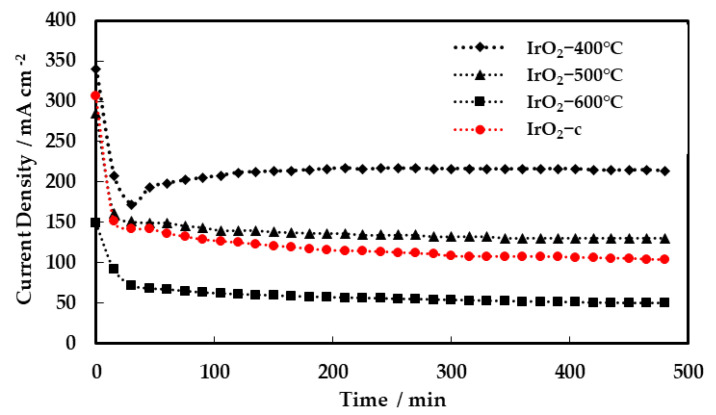
Current response of single cell during 8 h of continuous operation at 1.8 V with IrO_2_-400 °C, IrO_2_-500 °C, IrO_2_-600 °C, and IrO_2_-c as anodic electrocatalysts.

In order to evaluate the stability of the MEAs, short-term testing was carried out for 8 h continuous operation under constant applied potential (1.8 V), and the results are summarized in Figure 8.

The current density for all MEAs decreased quickly during the first minutes (~50 min) of potential application and then reached a steady-state value during the remaining time of testing. The initial drop in current is a common feature of MEAs subjected to a constant voltage (or current) switch. This rapid drop occurring during the first ~10 min of potential application (stabilization period) could be possibly ascribed to two main phenomena occurring under electrolysis conditions, i.e., a mass-transfer polarization or a modification of the oxidation state at the anode surface [29]. The best-performing MEA, with IrO_2_-400 °C as anode electrocatalyst, showed remarkable stability reaching 200 mA cm^−2^ after the initial stabilization period. These results are very promising regarding the stability of the synthesized electrocatalysts and MEAs.

## 3. Experimental Section

### 3.1. Catalyst Synthesis

A series of unsupported nanoparticle iridium oxide electrocatalysts were synthesized using a modified Adams method [2,3]. Firstly, the metal precursor H_2_IrCl_6_.xH_2_O (99 wt%, Alfa Aesar, USA) was dissolved in isopropanol with an excess of NaNO_3_ (Alfa Aesar, fine powder), and a homogeneous mixture was prepared. The metal concentration in the solution was 0.07 M. The salt mixture was heated at 60 °C under continuous stirring until isopropanol evaporated and the mixture was rather dry. Following, the mixture was allowed to dry completely for 30 min in an oven at 80 °C until a dry salt mixture was obtained. This dry salt mixture was then finally introduced into a ceramic furnace and sintered at 400, 500, and 600 °C for 30 min. After cooling to room temperature, the mixture was washed with distilled water in order to remove all Cl^−^ ions and remaining solvable salts, and finally dried in air at 80 °C overnight [9]. In the end, 0.2 g of catalyst was prepared.

### 3.2. Physicochemical Characterization

The crystalline structures of the synthesized electrocatalysts were analyzed by X-ray diffraction (XRD) using a Siemens D500 X-ray diffractometer with auto divergent slit and graphite monochromator using KaCu radiation (1.5418 Å) having a scanning speed 1.2° min^−1^. Τhe data were collected for 2θ values between 20 and 80°. The characteristic reflection peaks (d-values) were matched with JCPDS data files, and the crystalline phases were identified. The average crystallite size was calculated by means of the Scherrer formula after Warren’s correction for instrumental broadening. The unit cell parameters were refined with the CrystalSleuth (version: 19 May 2008) software.

The specific surface areas and pore volumes of the catalysts were determined using an Autosorb-1, Quantachrome instrument. The specific surface area was evaluated using the Brunauer–Emmett–Teller (BET) method.

The morphology, particle sizes, and microstructure of the metal oxides were studied with transmission electron microscopy (TEM). TEM images were obtained on a JEOL JEM 2010 high-resolution transmission electron microscope coupled with an Oxford INCA X-ray EDS for elemental analysis and surface mapping of the catalyst surface. Initial TEM images of the catalysts were collected at different regions.

The surface composition and metal oxidation state of the electrocatalysts were evaluated by X-ray photoelectron spectroscopy (XPS). The photoemission experiments were carried out in a commercial ultra-high vacuum system (*p* < 10^−9^ mbar), which consists of a fast-entry specimen assembly, sample preparation, and an analysis chamber. The analysis chamber is equipped with a SPECS LHS-10 hemispherical electron analyzer and a twin-anode X-ray gun, operating under the following conditions: unmonochromatized MgKa radiation at 1253.6 eV, analyzer pass energy of 97 eV, imposing a full width at half maximum (FWHM) of 1.7 eV for the Au 4f_7/2_ peak. The accuracy for binding energies assignments is ~0.1 eV, while in all samples, the main C 1s peak was at 284.6 eV. The XPS core level spectra were analyzed with a fitting routine, which decomposes each spectrum into individual, mixed Gaussian–Lorentzian peaks using a Shirley background subtraction over the energy range of the fit.

### 3.3. Electrochemical Experiments

Electrochemical experiments were performed using a standard three-electrode electrochemical cell in helium-purged 0.5 M H_2_SO_4_ solution to ensure the removal of diluted oxygen at 25 °C, using an Autolab PGSTAT302N potentiostat. A 3.5 mol L^−1^ KCl Ag/AgCl and Pt net were used as the reference and counter electrode, respectively, while the working electrode substrate was a glassy carbon disk (rotating disk electrode, disc area: 0.07065 cm^2^). The working electrode consisted of a catalyst layer loading 3.25 mg cm^−2^. This layer was prepared by dispersing 10 mg of the catalyst powder in 1 mL of ethanol, and 80 μL of Nafion solution (5 wt%, Sigma Aldrich, Burlington, MA, USA) was also added to the catalyst ink solution as the binder. After that, the catalyst ink solution was sonicated for 30 min at room temperature to ensure uniform dispersion, and consequently, an amount of 25 μL was cast on the clean, glassy carbon substrate using a micropipette. The formatted electrode was dried in air for 1 h.

Ιnitially, the prepared ΙrO_2_ catalysts were characterized by repetitive cyclic voltammetry (CV) in the three-electrode cell between 0 and 1.45 V vs. RHE with scan rates of 20, 50, 75, and 100 mV s^−1^. The electrochemically active surface areas (ECSAs) were assumed to be proportional to the anodic redox charge of the CVs, as proposed by Ardizone et al. [30]. Here the results were obtained by integration of the anodic voltammetric profiles at various sweep rates.

The OER activities of the catalysts were evaluated by linear sweep voltammetry (LSV) within the potential range between 1.30 and 1.70 V_RHE_ at a scan rate of 1 mV s^−1^ (near steady state). To avoid the accumulation of oxygen bubbles, a rotation speed of 2500 rpm was applied using a Metrohm rotator system. The electrolyte was deaerated via bubbling with He prior to and during all measurements. All reported measurements were repeated at least three times to ensure the reproducibility of the results.

### 3.4. Preparation of Membrane Electrode Assemblies (MEAs)

A Nafion^®^115 (DuPont^TM^, Wilmington, NC, USA) membrane with a typical thickness of 127 μm was used as a solid polymer electrolyte. IrO_2_ oxides powders, prepared as described above, were used as OER electrocatalyst while Pt/C catalyst (BASF, 20% Pt on Vulcan XC-72 A65TDV 2.1 ELAT^®^ V2.1) was used as hydrogen evolution electrocatalyst at the cathode. Electrodes were prepared as follows: catalysts and Nafion ionomer (5 wt%, Sigma Aldrich, Burlington, MA, USA) mixtures were first ultrasonically suspended in a mixture of deionized water and isopropanol. Catalytic suspensions thus obtained were then brushed directly onto the Nafion^®^115 membrane (Catalyst Coated Membrane, CCM). The catalyst loadings were 0.5 mg cm^−2^ Pt/C for the hydrogen side and 1.5 mg cm^−2^ IrO_2_ for the oxygen side. Electrode layers (with 25 cm^2^ active area) were bonded by placing the catalyst-coated membrane between carbon paper for the oxygen side and carbon cloth for the hydrogen side and then by hot pressing at 130 °C and 10 MPa for 3 min. The Nafion content of the electrocatalytic layers was set to 30 wt% for the anode. Finally, the single-cell tests were carried out with a PEM water electrolysis cell. MEAs were clamped between a porous carbon paper at the anode and a carbon cloth as a gas diffusion layer at the cathode side. The cell body was made of end plates (stainless steel), graphite bipolar plates with serpentine flow fields (suitable for electrolysis), and the MEA between them. The cell was tightened between two stainless steel plates, using a dynamometric wrench to set the fastening screws to 40 cNm.

## 4. Conclusions

IrO_2_ catalysts were synthesized by the modified Adams method. The effect of the calcination temperature, in the range between 400 and 600 °C, on the morphological/surface characteristics and the electrochemical performance of the catalysts for the oxygen evolution reaction (OER) was studied.

The application of a modified Adams method resulted in the synthesis of unsupported nanostructured IrO_2_ electrocatalysts with small, nano-scale particle size (1.24–7.77 nm) and high BET surface area (185–66 m^2^g^−1^). The structures, activities, and stabilities of Adams’ fusion method prepared iridium oxide nanomaterials as catalysts for the OER were studied. Heat treatment at different temperatures provided IrO_2_ catalysts with varying degrees of crystallinity. The unsupported prepared materials exhibit outstanding activity for oxygen evolution reaction (OER) compared to commercial IrO_2_. To achieve both high OER activity and stability, three key factors have been identified: (i) high anodic charge, (ii) high surface area due to nano-size IrO_x_ particles well dispersed in the Nafion ionomer electrolyte, and (iii) homogeneous layer morphology. The best-performing material was the IrO_2_ calcined at 400 °C.

The performances of the unsupported catalysts were also studied in MEA configuration under practical water electrolysis conditions. In agreement with the OER activity measurements, the best-performing MEA was the one employing the 400 °C calcined IrO_2_ catalyst. In terms of stability, the tests of four MEAs at steady operation stability (1.8 V) after 8 h continuous operation perform excellent stability. Further studies are required to examine the long-term (in the range of 1000–10,000 h) performance of the catalyst in electrolyzer conditions.

## Figures and Tables

**Table 1:** Results of the XRD analysis.

Electrocatalyst	Lattice Parameters		Crystallite Size	Unit Cell Volume
			nm	Å^3^
IrO_2_-c	-	-	1.24 *	-
IrO_2_-400 °C	-	-	1.40 *	-
IrO_2_-500 °C	a = b = 4.467	c = 3.110	5.18	62.0
IrO_2_-600 °C	a = b = 4.457	c = 3.140	7.77	62.4

* Estimated from TEM images.

**Table 2:** BET surface, pore volume, and total ESCA obtained for IrO_2_ electrocatalysts calcined at different temperatures.

Electrocatalyst	Surface Area	Pore Volume	ESCA
	m^2^ g^−1^	cm^3^ g^−1^	m^2^ g^−1^
IrO_2_-c	32	0.130	11
IrO_2_-400 °C	185	0.200	198
IrO_2_-500 °C	127	0.167	27
IrO_2_-600 °C	66	0.145	21

**Table 3:** Tafel slopes of ΙrO_2_-400 °C, ΙrO_2_-500 °C, ΙrO_2_-600 °C, and ΙrO_2_-c electrocatalysts.

	Tafel Slope/mV dec^−1^	
Electrocatalyst	Low Overpotentials	High Overpotentials
IrO_2_-c	60	118
IrO_2_-400 °C	61	115
IrO_2_-500 °C	110	120
IrO_2_-600 °C	113	157

## Data Availability

Data is contained within the article.
